# Kinetics and Novel Degradation Pathway of Permethrin in *Acinetobacter baumannii* ZH-14

**DOI:** 10.3389/fmicb.2018.00098

**Published:** 2018-02-02

**Authors:** Hui Zhan, Huishan Wang, Lisheng Liao, Yanmei Feng, Xinghui Fan, Lianhui Zhang, Shaohua Chen

**Affiliations:** State Key Laboratory for Conservation and Utilization of Subtropical Agro-bioresources, Guangdong Province Key Laboratory of Microbial Signals and Disease Control, Integrative Microbiology Research Centre, South China Agricultural University, Guangzhou, China

**Keywords:** permethrin, degradation pathway, kinetics, *Acinetobacter baumannii*, bioremediation

## Abstract

Persistent use of permethrin has resulted in its ubiquitous presence as a contaminant in surface streams and soils, yet little is known about the kinetics and metabolic behaviors of this pesticide. In this study, a novel bacterial strain *Acinetobacter baumannii* ZH-14 utilizing permethrin via partial hydrolysis pathways was isolated from sewage sludge. Response surface methodology based on Box-Behnken design of cultural conditions was used for optimization resulting in 100% degradation of permethrin (50 mg·L^−1^) within 72 h. Strain ZH-14 degraded permethrin up to a concentration of 800 mg·L^−1^. Biodegradation kinetics analysis indicated that permethrin degradation by this strain was concentration dependent, with a maximum specific degradation rate, half-saturation constant, and inhibition constant of 0.0454 h^−1^, 4.7912 mg·L^−1^, and 367.2165 mg·L^−1^, respectively. High-performance liquid chromatography and gas chromatography-mass spectrometry identified 3-phenoxybenzenemethanol and 3-phenoxybenzaldehyde as the major intermediate metabolites of the permethrin degradation pathway. Bioaugmentation of permethrin-contaminated soils with strain ZH-14 significantly enhanced degradation, and over 85% of permethrin was degraded within 9 days with the degradation process following the first-order kinetic model. In addition to degradation of permethrin, strain ZH-14 was capable of degrading a large range of synthetic pyrethroids such as deltamethrin, bifenthrin, fenpropathrin, cyhalothrin, and beta-cypermethrin which are also widely used pesticides with environmental contamination problems, suggesting the promising potentials of *A. baumannii* ZH-14 in bioremediation of pyrethroid-contaminated terrestrial and aquatic environments.

## Introduction

Synthetic pyrethroids (SPs) are the chemical analogs of pyrethrins, which are natural compounds extracted from the flowers of *Chrysanthemum cinerariaefolium* (Cycoń and Piotrowska-Seget, 2016). The mechanism of pyrethroid action is affecting the sodium channels of neurons (Wang et al., 2016). SPs are commonly considered to be safer than organophosphate and carbamate insecticides for their low mammalian and avian toxicity (Tu et al., 2016). However, the extensive use of these compounds in a wide variety of fields has resulted in widespread contamination of the environment that is of ecological concern (Cycoń and Piotrowska-Seget, 2016). Increasing evidence indicated that SPs may negatively affect non-target organisms, for instance, SPs are enormously toxic to aquatic organisms and bees (Tu et al., 2016), some SPs are harmful to the nervous system, immune system, and endocrine system of human beings (Ratelle et al., 2015).

**Figure 1:**
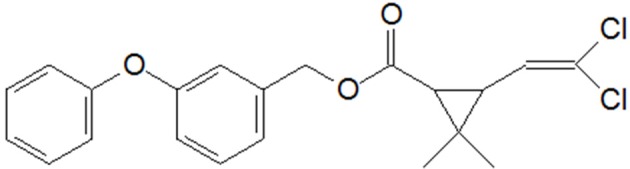
Molecular structure of permethrin.

Permethrin (3-phenoxybenzyl 3-(2,2-dichlorovinyl)-2,2-dimethylcyclopropanecarboxylate) (Figure 1), belonging to the Type I SPs, has been frequently and extensively used in agriculture and residential home. Numerous reports revealed that the residue of permethrin is ubiquitous in surface water via various approaches entering the aquatic environments (Delgado-Moreno et al., 2011; Shahsavari et al., 2012; Yan et al., 2012; Mozhdeganloo et al., 2016). Moreover, permethrin is suspected to disorder the neurons of children and adults as a consequence of chronic exposure to it (Willemin et al., 2015). The increasing use of permethrin may pose great risks to terrestrial and aquatic environments as well as public health.

It is urgent to find out cost-effective and eco-friendly ways to reduce the environmental and public health risks associated with pyrethroid use. Various remediation technologies including oxidation, photo degradation, adsorption, and bacterial degradation have been developed for this purpose (Lin et al., 2011; Yang et al., 2011; Gao et al., 2012); however, bioremediation which is based on the catabolic activity of pesticide-degrading bacteria, has emerged as the most promising strategy for cleaning-up pyrethroid-contaminated environment (Cycoń and Piotrowska-Seget, 2016; Arora et al., 2017). Thus, far, many bacterial strains have been isolated to degrade SPs, including *Ochrobactrum anthropi* YZ-1 (Zhai et al., 2012), *Brevibacterium aureum* DG-12 (Chen et al., 2013), *Catellibacterium* sp. CC-5 (Zhao et al., 2013), *Serratia marcescens* (Cycoń et al., 2014), *Acinetobacter calcoaceticus* MCm5 (Akbar et al., 2015), *Bacillus thuringiensis* ZS-19 (Chen et al., 2015), and *Pseudomonas aeruginosa* GF31 (Tang et al., 2017). However, little is known about the kinetics and metabolic behaviors of permethrin. Moreover, the bioremediation potentials of permethrin-contaminated soils using isolated strains have not been investigated so far and the degradation mechanism remains unknown.

The objectives of the present study were to isolate and identify an efficient degrader for bioremediation of permethrin-contaminated environment, to determine the degradation kinetics of permethrin and other SPs, and to elucidate the biodegradation mechanism of permethrin by the strain.

## Materials and methods

### Chemicals reagents and media

Technical-grade permethrin, deltamethrin, bifenthrin, fenpropathrin, cyhalothrin, and beta-cypermethrin were obtained from Sigma-Aldrich, USA. Chromatographic grade acetonitrile was purchased from Fisher Scientific, USA. All other reagents used here were obtained from Sigma-Aldrich, USA. The chemicals were dissolved in acetone or dimethyl sulfoxide (DMSO) at a stock concentration of 10 g·L^−1^, and stored in dark bottles at 4°C prior to use.

The mineral salt medium (MSM) containing 2 g (NH_4_)_2_SO_4_, 0.2 g MgSO_4_·7H_2_O, 0.01 g CaCl_2_·2H_2_O, 0.001 g FeSO_4_·7H_2_O, 1.5 g Na_2_HPO_4_·12H_2_O, and 1.5 g KH_2_PO_4_ per liter was used for biodegradation assays in the study. The Luria-Bertani medium (LB) containing 10 g tryptone, 5 g yeast extract and 10 g NaCl per liter was used for incubating *Acinetobacter baumannii* ZH-14. Both of the media were autoclaved at 121°C for 20 min. The pH of the media was adjusted to 7.0.

### Enrichment, isolation, and screening of permethrin degraders

The sewage sludge samples used to isolate the permethrin degraders were collected from the wastewater pond located in Singapore. Approximately 5 g of the sample was added to a 250-mL Erlenmeyer flask containing 50 mL of sterilized MSM enrichment medium and 50 mg·L^−1^ of permethrin was dissolved in acetone as the final concentration. Then the enrichment culture was incubated at 30°C in a rotary shaker at 180 rpm for 7 days. Afterwards, 5 mL of enrichment culture was transferred into 50 mL of fresh MSM medium containing 100 mg·L^−1^ of permethrin for another 7-day incubation. The enrichment culture was successively transferred into fresh enrichment medium containing 200, 400, and 800 mg·L^−1^ of permethrin for another 7-day incubation as the previous operation. The final culture was serially diluted and spread on MSM agar (1.8%) plates containing permethrin (50 mg·L^−1^) for incubation at 30°C for 5 days. The individual colonies that utilized permethrin as sole carbon and energy source for growth were selected, picked, and purified by re-streaking three times (Chen et al., 2015). The ability of isolates to degrade permethrin was determined by high-performance liquid chromatography (HPLC) (Waters, USA) as described below. One pure isolate designated ZH-14 showing the highest degradation activity was selected for further study.

### Bacterial identification

The isolate was characterized and identified by morphology, physio-biochemical characteristics, 16S rRNA gene analysis as well as Biolog Microbial Identification System. Following the described method (Deng et al., 2015), the 2-day colonies of strain ZH-14 were observed under a light microscope (Olympus, Japan) like size, color, surface, edge, texture. To further understand the morphology, cells were examined by scanning electron microscopy (SEM) (Hitachi, Japan). Physio-biochemical assays were tested with reference to Bergey's Manual of Determinative Bacteriology (Holt et al., 1994).

Total genomic DNA was extracted with a MasterPure™ DNA Purification Kit (Epicentre Biotechnologies, USA) according to the protocols of the manufacturer. The 16S rRNA gene was PCR-amplified with universal primer pairs, B1 (5′-TGACGAGTGGCGGACGGGTG-3′) and B4 (5′-CCATGGTGTGACGGGCGGTGTG-3′). The resulting sequences of PCR products (1,205 bp) were compared with the genes available in the Genbank Nucleotide Library through a BLAST search in the National Center for Biotechnology Information (NCBI). Multiple alignments of 16S rRNA were performed using CLUSTALX 1.8.1, and phylogeny was analyzed with MEGA 4.0 (Tamura et al., 2007). The unrooted tree was constructed by neighbor-joining method. Finally, the bacterium was further identified by Biolog Microbial Identification System (Microstation, USA) according to the instructions of the manufacturer.

### Optimization of cultural conditions

Response surface methodology (RSM) based on the Box-Behnken design was used to optimize the crucial factors and interactive influences which remarkably affect the permethrin degradation by ZH-14. According to the result of preliminary one-factor-at-a-time experiments, three main factors including pH, temperature, and inocula were selected as independent variables to obtain optimal conditions for permethrin degradation (Chen et al., 2013). The dependent variable was the degradation of 50 mg·L^−1^ permethrin in MSM for 5 days. A three-variable Box-Behnken design consisting of 15 experimental runs with three replicates at the midpoint was generated by statistic analysis system (SAS) software (Version 9.2, SAS Institute Inc., Cary, NC, USA; Table S1). The data was analyzed through response surface regression procedure to fit the following quadratic polynomial equation (Equation 1; Chen et al., 2013).

(1) $$\begin{array}{l} Y_{i}=b_{0}\;+\;\sum \;b_{i}X_{i}\;+\;\sum \;b_{ij}X_{i}X_{j}\;+\;\sum \;b_{ii}X_{i}^{2} \end{array}$$

Where *Y*_*i*_ is the predicted response, *X*_*i*_ and *X*_*j*_ are variables, *b*_0_ is the constant, *b*_*i*_ is the linear coefficient, *b*_*ij*_ is the interaction coefficient, and *b*_*ii*_ is the quadratic coefficient.

### Growth and degradation assays

*A. baumannii* strain ZH-14 was stored in 20% glycerol at −80°C. Before each experiment the bacterial strain was thawed and grown in 250-mL Erlenmeyer flasks containing 50 mL of sterile LB medium. Then the cultures were incubated at 30°C in a rotary shaker at 180 rpm. The bacterial cells in the late-exponential growth phase were harvested by centrifugation (5 min, 4000 rpm) at 4°C and washed twice in 0.9% normal saline (*N*-saline) before inoculation (Chen et al., 2011a). The density of strain ZH-14 was adjusted with sterile *N*-saline to ~1.0 × 10^8^ CFU·mL^−1^, unless otherwise stated. One percent of the resuspension was used as the inocula for the subsequent studies.

Strain ZH-14 was incubated in MSM culture at 30°C and 180 rpm on a rotary shaker for 72 h, utilizing 50 mg·L^−1^ of permethrin as the sole carbon source, with a non-inoculated culture served as a control. All experiments were carried out in triplicate under the optimal degradation conditions. Culture samples were collected regularly at an interval of 12 h (0, 12, 24, 36, 48, 60, 72 h) from the cultures. The growth of strain ZH-14 was monitored by measuring the optical density (OD) at 600 nm by UV-spectrophotometer. The amount of residual permethrin was determined by HPLC as described below.

### Degradation kinetics of permethrin and 3-phenoxybenzenemethanol

Degradation kinetic experiments with different initial permethrin concentrations (25, 50, 100, 200, 400, 600, and 800 mg·L^−1^) by strain ZH-14 were performed at 30°C and 180 rpm on a rotary shaker. 3-Phenoxybenzenemethanol is a major metabolite from the degradation of SPs, which in turn had the effects of antimicrobial activities thus accelerated degradation of SPs could not occur (Laffin et al., 2010). To study the effect of initial 3-phenoxybenzenemethanol concentration on biodegradation, strain ZH-14 was incubated in MSM supplemented with different concentrations of 3-phenoxybenzenemethanol (25, 50, 100, 200, 300, 400, and 500 mg·L^−1^). All the cultures were carried out in triplicate with non-inoculated cultures as controls. Culture samples were collected at regular intervals and centrifuged. The residue of chemical was measured by HPLC.

### Identification of permethrin metabolites

Strain ZH-14 was grown in 250-mL Erlenmeyer flask with 50 mL of sterilized MSM containing different concentrations of permethrin (50, 100, and 200 mg·L^−1^) as the sole source of carbon and energy at 30°C and 180 rpm for 72 h. The same culture without permethrin was served as a negative control, and non-inoculated culture was used as a positive control. The metabolites of permethrin of strain ZH-14 cultures were extracted and identified by chromatography-mass spectrometry (GC-MS) (Agilent, USA) following the method of Tallur et al. (2008) with modification. In brief, samples were collected at an interval of 12 h. After centrifuging for 20 min, the supernatant was adjusted to pH 2 with 2 M HCl, extracted with ethyl acetate and filtrated with 0.45 μm membrane. Finally, the samples were analyzed by GC-MS as described below.

### Degradation kinetics of various SPs

To study its ability to degrade various SPs, strain ZH-14 was inoculated in sterilized MSM added with 50 mg·L^−1^ of permethrin, deltamethrin, bifenthrin, fenpropathrin, cyhalothrin, and beta-cypermethrin, respectively. All cultures were conducted in triplicate with non-inoculated cultures served as controls. The sampling was carried out at a 12-h time interval and the amount of residual pesticides was measured by HPLC as described below.

### Biodegradation of permethrin in soils

The soil samples were collected from the top layer of 0–20 cm in a farmyard where permethrin and fertilizers had not been used for 5 years, located in South China Agricultural University, Guangdong Province, China. The detailed physicochemical properties of the soil were (grams per kilogram of dry weight): organic matter, 10.5; total N, 0.5; total P, 0.4; total K, 18.2; and pH, 6.9. The soils have a sandy loam texture (sand, 65.0%; silt, 28.0%; clay, 7.0%; Chen et al., 2014). The moisture degree of soil was kept as a constant level which was suitable for use.

To investigate the degradation kinetics of permethrin in soils, strain ZH-14 was inoculated in sterile and nonsterile soils and incubated in a darkened thermostatic chamber at 30°C for 9 days according to our previous method with slight modification (Chen et al., 2012, 2014). Briefly, 50 mg·L^−1^ final concentration of permethrin solution was sprayed on the surface of 500 g of sterile and nonsterile soils in 500-mL Erlenmeyer flask. After thorough mixing, the bacterial suspension was introduced into the soil samples at a final bacterial count of ~1.0 × 10^8^ CFU·g^−1^. The triplicate samples of sterile and nonsterile soils lacking strain ZH-14 were served as controls, respectively. All soil samples maintain ~40% of water-holding capacity by adding sterile deionized water. Soil samples (20 g) were collected aseptically from 500-mL Erlenmeyer flask at 1st, 3rd, 5th, 7th, and 9th day for the measurement of permethrin residues.

### Chemical analysis

Chemical quantification was monitored with a Waters 2690 HPLC system equipped with a ternary gradient pump, programmable variable-wavelength UV detector, column oven, electric sample valve, and C_18_ reversed-phase column (Phenomenex Lunar 5 μm C_18_ 250 × 4.6 mm) with array detection from 190 to 400 nm (total scan) based on retention time and peak area of the pure standard. The samples were determined using a mobile phase of 70:30 acetonitrile/water at a flow rate of 1.0 mL·min^−1^. The injection volume was 10 μL (Chen et al., 2015).

The metabolites of permethrin were analyzed and identified with an Agilent 6890N/5975 GC-MS system equipped with an autosampler and OnColumn, split/splitless capillary injection system, with an HP-5MS capillary column (30.0 m × 250 μm × 0.25 μm) with array detection from 30 to 500 nm (total scan). The operating conditions were following the method of our previous study (Chen et al., 2014). The ionization energy was 70 eV, and the temperatures of the transfer line and the ion trap were 280 and 230°C, respectively. The injection volume was 1.0 μL with splitless sampling at 250°C. High purity helium gas was used as a carrier gas at a flow rate of 1.5 mL min^−1^.

### Kinetic analysis

The substrate inhibition model [Equation (2)] adapted from Luong (1987) was used to fit the specific degradation rate (*q*) at different initial concentrations of permethrin or 3-phenoxybenzenemethanol.

(2) $$\begin{array}{l} q=\frac{q_{\max}S}{S+K_{s}+\left(S^{2}/K_{i}\right)} \end{array}$$

where *q*_max_ is the maximum specific degradation rate, *K*_i_ is the substrate inhibition constant, *K*_s_ is the half-saturation constant, and *S* is the inhibitor concentration. The *q* value was calculated from the gradient of a semi-logarithm plot of permethrin or 3-phenoxybenzenemethanol concentration.

Degradation process of various SPs in liquid media or soils was fitted to the first-order kinetic model (Equation 3) adapted from Cycoń et al. (2014).

(3) $$\begin{array}{l} C_{t}=C_{0}\;\times \;e^{-kt} \end{array}$$

where *C*_0_ is the initial concentration of substrate, *C*_t_ is the amount of substrate at time *t, k* is the degradation rate constant, and *t* is the degradation time.

The theoretical half-life (*t*_1/2_) values of different SPs were calculated by Equation 4.

(5) $$\begin{array}{l} t_{1/2}=\frac{\ln\;\left(2\right)}{k} \end{array}$$

where ln (2) is the natural logarithm of 2, and *k* is the degradation rate constant.

## Results and discussion

### Isolation and characterization of strain ZH-14

As the result of enrichment and isolation, one isolate designated as ZH-14 utilizing permethrin as the sole source of carbon and energy was selected for further study. Strain ZH-14 degraded ~92.4% of permethrin within 48 h of incubation. It was characterized as Gram-negative by Gram staining, and observed as rod-shaped by SEM with dimensions of 13–24 μm in length and 6–9 μm in width (Figure S1). The optimum temperature for growth is 30°C, able to grow at 40°C, pH range for growth is 5–10, could grow with 0–5% NaCl in LB broth. Its colonies on LB plate are beige, round, convex, glossy, and smooth with entire margins. It was positive in tests or reactions such as oxidase, catalase, hemolysis, and citrate while it was negative in utilization of nitrate, urease, indole, gelatin liquefaction, esculin hydrolysis, lysine decarboxylase, and ornithine decarboxylase (Table 1). Further biochemical results tested by Biolog Microbial Identification System are showed in Table S2.

Table 1Morphological and physio-biochemical characteristics of strain ZH-14.

**Table:** 

**Characteristics**	**Results**	**Characteristics**	**Results**
Colonies	Beige, round, convex, glossy, and smooth with entire margins	Cells	Rod-shaped, 13 to 24 μm in length and 6 to 9 μm in width
Liquid culture	Turbid liquid	Gram staining	Negative
Catalase	+	Oxidase	+
Citrate	+	Nitrate	−
Urease	−	Indole	−
Gelatin liquefaction	−	Esculin hydrolysis	−
Ornithine decarboxylase	−	Lysine decarboxylase	−
Hemolysis	+	Anaerobic	−

**Table:** 

Temperature	30°C	40°C	50°C	60°C
	+	+	−	−
Salt endurance	2%NaCl	5%NaCl	7%NaCl	10%NaCl
	+	+	−	−

**Table:** 

pH	3	5	7	9	10
	−	+	+	+	+

*+, tested positive/utilized as substrate; −, tested negative/utilized as substrate*.

**Figure 2:**
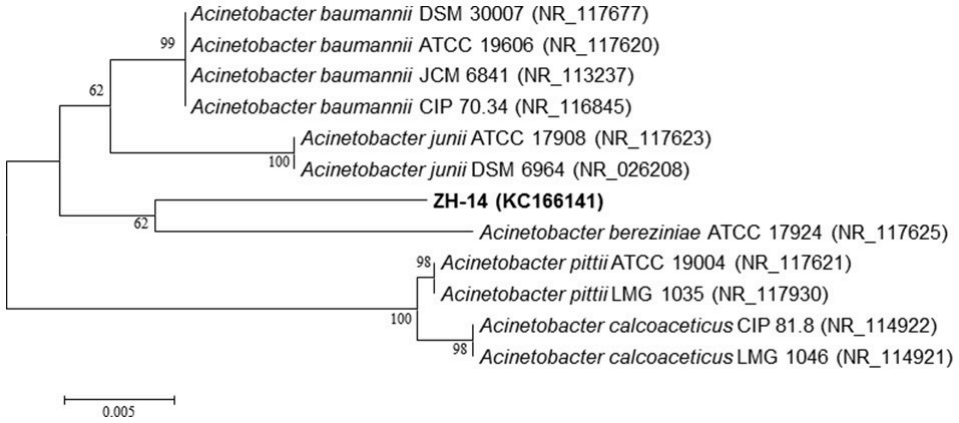
Phylogenetic analysis based on the 16S rRNA sequence of strain ZH-14 and other representative *Acinetobacter* strains. The phylogenetic tree was constructed with the neighbor joining method (NJ). Numbers in parentheses represent the sequences accession number in GenBank. Numbers at the nodes indicate bootstrap values from the neighborhood-joining analysis of 1,000 resampled data sets. Bar represents sequence divergence.

Analysis of the 16S rRNA gene sequence revealed that strain ZH-14 belongs to the genus of *Acinetobacter*, showing high similarities to *A. baumannii* (99%). The phylogenetic relationships of the 16S rRNA gene sequences of strain ZH-14 and other representative *Acinetobacter* strains are shown in Figure 2. The draft genome sequence of strain ZH-14 was submitted to the GenBank under accession number KC166141. Based on the morphology, physio-biochemical characteristics, and 16S rRNA gene analysis as well as Biolog tests, strain ZH-14 was identified as *A. baumannii*.

Many bacteria have been isolated and characterized with their ability to transform various xenobiotic compounds, and the bacteria from genera *Bacillus* and *Pseudomonas* are the most metabolically active microorganisms (Arora et al., 2014; Arora, 2015; Cycoń and Piotrowska-Seget, 2016). *A. baumannii* is a widespread bacterium in various natural habitats and possesses broad catabolic capabilities, and it was found to be highly effective in degrading diesel-oil (Nkem et al., 2016), congo red (Li et al., 2015), and 1,4-dioxane and BTEX mixtures (Zhou et al., 2016). Nevertheless, the potential use of *A. baumannii* in bioremediation of environmental pollutants has not received the attention it deserves. Furthermore, reports on pyrethroid-degrading isolates from *A. baumannii* are scarce in the literature. This study provides the first evidence that *A. baumannii* participates in efficient degradation of permethrin and other SPs.

### Growth and degradation assays with strain ZH-14

**Figure 3:**
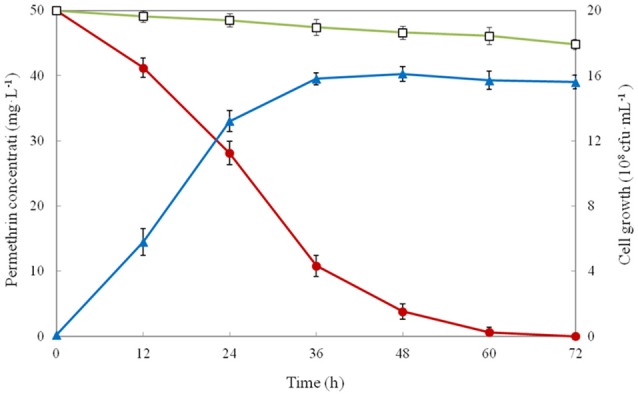
Biodegradation of permethrin in MSM by strain ZH-14. Symbol: □, permethrin control; •, permethrin biodegradation; ▴, cell growth. Data represent mean values of three replicates with standard deviation.

The growth and degradation of strain ZH-14 were investigated in MSM using 50 mg·L^−1^ of permethrin as the sole carbon source. As the result showed that growth of strain ZH-14 was accumulated without lag phase indicating that strain ZH-14 was able to utilize permethrin as growth substance (Figure 3). Meanwhile, compared with the control, the degradation of permethrin was demonstrably facilitated in the presence of strain ZH-14. Permethrin degradation was dependent on bacterial cell density, and the biodegradation rate of permethrin increased rapidly at logarithmic phase and slowed down at stationary phase. Approximately 92.4 and 98.8% of the permethrin was degraded by strain ZH-14 in 48 and 60 h, respectively. Finally, the residual amount of permethrin was not detectable by HPLC at 72 h post incubation. In contrast, there was no significant change in permethrin concentration in the non-inoculated controls. To our knowledge, this is the first report that a bacterial isolate completely degraded permethrin. To date, few strains have been reported to degrade permethrin, for instance, *Sphingobium* sp. JZ-2 was capable of degrading nearly 90% of permethrin (50 mg·L^−1^) within 5 days (Guo et al., 2009). One recently isolated pyrethroid-degrading strain, *Bacillus* sp. DG-02, could degrade 63.6% of the added permethrin (50 mg·L^−1^) within 72 h (Chen et al., 2014). It is noteworthy that isolates were usually unable to grow or transform SPs in the absence of carbon source during the catabolism of these insecticides (Maloney et al., 1988). Strain ZH-14 could utilize permethrin as the sole source of carbon for growth, and it is more effective than other isolated bacteria in degrading SPs, revealing the promising potentials of *A. baumannii* ZH-14 in bioremediation of pyrethroid-contaminated environment.

### Optimization of culture conditions for permethrin degradation

Based on preliminary results of one-factor-at-a-time experiments, three independent variables including temperature (*X*_1_), pH (*X*_2_), and inocula (*X*_3_) were manipulated and optimized for enhancement of permethrin biodegradation using RSM (Zhang et al., 2011; Chen et al., 2013). The data obtained for the residual amount of permethrin (*Y*_1_) are representing the combined effect of these three variables at various levels. The experimental design variables corresponding to the responses for permethrin residues are presented in Table S1. The data were analyzed by SAS software containing response surface regression procedure. The experimental values of permethrin residues were fitted with the following quadratic polynomial model equation [Equation (5)].

(5) $$\begin{array}{l} Y_{1}=478.1469-55.9875X_{1}-17.7825X_{2}-172.625X_{3}\\ +\;4.1125X_{1}^{2}-0.125X_{1}X_{2}+3.0X_{1}X_{3}+0.3125X_{2}^{2}\\ +\;1.7X_{2}X_{3}+258.75X_{3}^{2} \end{array}$$

where *Y*_1_ is the predicted permethrin residues; *X*_1_, *X*_2_, and *X*_3_ are the coded values of temperature, pH, and inocula, respectively.

Analysis of variance (ANOVA) for the fitted quadratic polynomial model is showed in Table 2. A *R*^2^ of 0.9912 indicated that most of the randomness in response could be covered by the model, implying that the predicted values of the model were consistent with the experimental values. The results of regression analysis revealed that linear and square terms of temperature (*X*_1_), pH (*X*_2_) and inocula (*X*_3_) all showed significant effects (*P* < 0.05) on the permethrin degradation by strain ZH-14. The response surface plots illustrated the interactive effects on permethrin degradation by strain ZH-14 (Figure S2). As seen in the Figure S2, at the stationary point, there was a minimum value of residual permethrin, which also represented a theoretical maximum value of permethrin degradation. The optimum levels for the three variables of *X*_1_, *X*_2_, and *X*_3_ were determined in terms of the coded units at the stationary point which is temperature 30°C, pH 7.0 and an inocula size of 0.2 g·L^−1^, respectively.

**Table 2:** Analysis of variance (ANOVA) for the fitted quadratic polynomial model.

**Source**	**DF**	**SS**	**MS**	***F* Value**	*P* Level^*^
*X*_1_	1	23.4613	23.4613	34.8866	0.0020
*X*_2_	1	24.1513	24.1513	35.9126	0.0019
*X*_3_	1	42.32	42.32	62.9294	0.0005
*X*_1_*X*_1_	1	62.4467	62.4467	92.8576	0.0002
*X*_1_*X*_2_	1	1.5625	1.5625	2.3234	0.1879
*X*_1_*X*_3_	1	0.36	0.36	0.5353	0.4972
*X*_2_*X*_2_	1	225.3606	225.3606	335.1087	0.0001
*X*_2_*X*_3_	1	2.89	2.89	4.2974	0.0929
*X*_3_*X*_3_	1	24.7206	24.7206	36.7592	0.0018
Model	9	377.1535	41.9059	62.3137	0.0001
Error	5	3.3625	0.6725		
Total	14	380.516			

*R^2^ = 0.9912. DF refers to degrees of freedom; SS refers to sum of sequences; MS refers to mean square*.

* *P Level < 0.05 indicates the model terms are significant*.

RSM is an empirical modeling system that has been widely used to improve and optimize complex processes, including biodegradation processes and fermentation processes for a variety of microorganisms (Moon et al., 2011; Zhang et al., 2011; Chen et al., 2013; Xiao et al., 2015). In this study, a quadratic polynomial model [Equation (2)] was successfully developed, resulting in improved yields of degradation by strain ZH-14 with fewer experiments and minimal resources. Temperature, pH and inocula size have a great influence on the biodegradation, consistent with previous findings of Zhang et al. (2011) and Cycoń et al. (2014). Additionally, it is noteworthy that strain ZH-14 maintained relatively high permethrin removal efficiency over a wide range of temperature and pH, with the maximal activity at 30°C and pH 7.0, implying its excellent environmental adaptation.

### Degradation kinetics of permethrin and 3-phenoxybenzenemethanol

**Figure 4:**
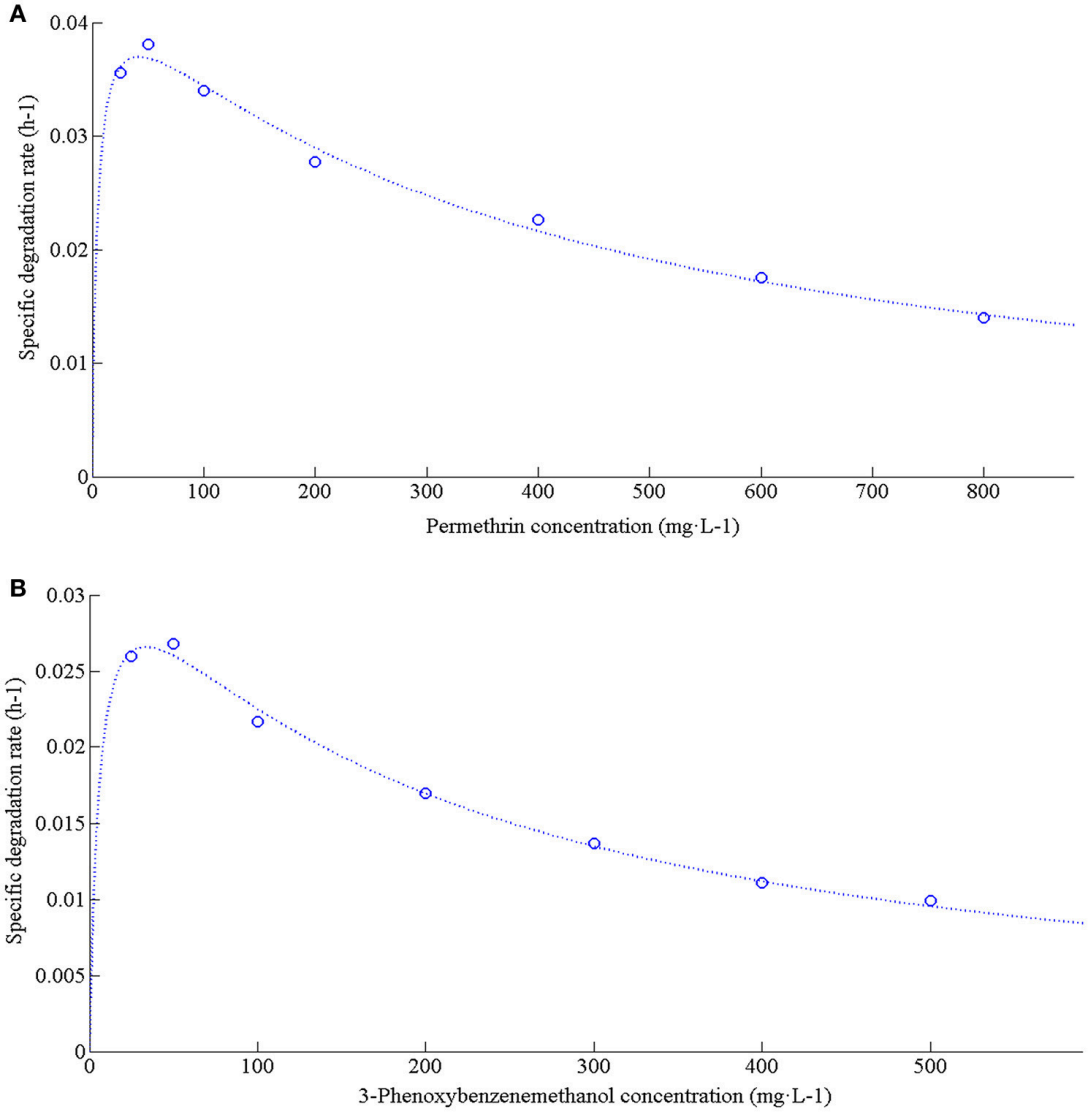
Degradation kinetics of permethrin and 3-phenoxybenzenemethanol with different initial concentrations by strain ZH-14. **(A)** Relationship between initial permethrin concentration and specific degradation rate by strain ZH-14; **(B)** Relationship between initial 3-phenoxybenzenemethanol concentration and specific degradation rate by strain ZH-14.

Strain ZH-14 rapidly degraded and utilized permethrin up to a concentration of 800 mg·L^−1^, and a lag phase was observed at higher permethrin concentrations (Figure 4A). According to the kinetics analysis, permethrin degradation by this strain was concentration dependent, so the substrate inhibition model [Equation (2)] adapted from Luong (1987) was used to fit the specific degradation rate (*q*) at different initial concentrations of permethrin.

**Figure 5:**
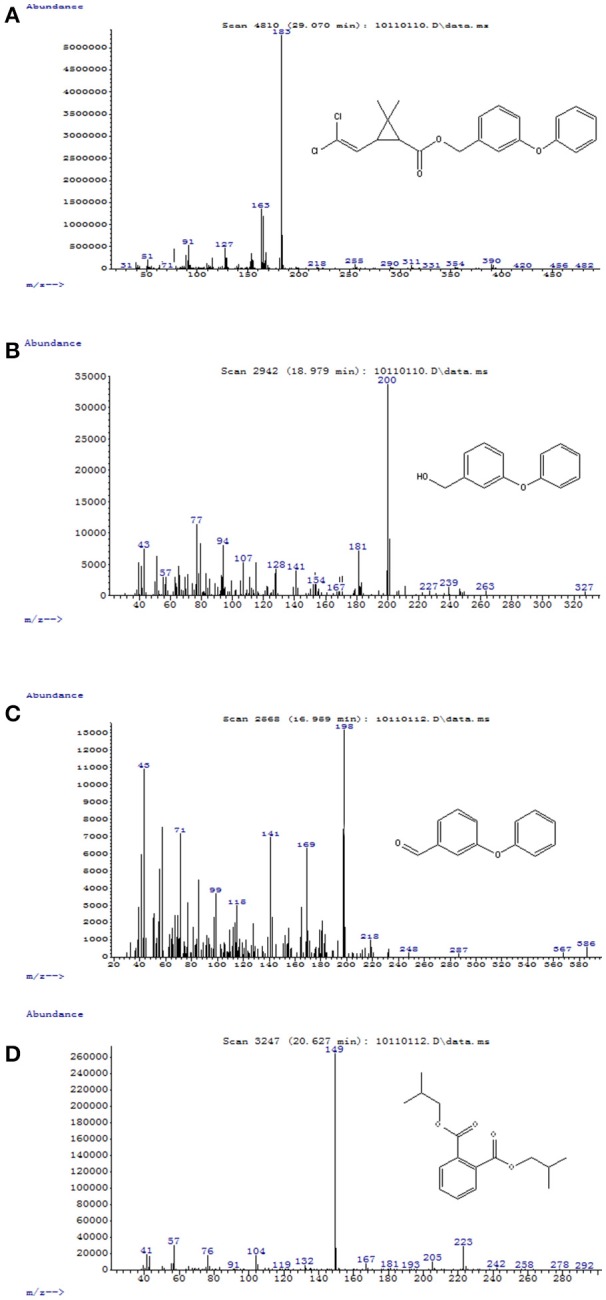
GC-MS analysis of the metabolites produced from permethrin degradation by strain ZH-14. **(A–D)** The characteristic ions of compounds **(A–D)**. The retention time of each compound was 29.070, 18.979, 16.959, and 20.627 min, respectively, which were identified as permethrin, 3-phenoxybenzenemethanol, 3-phenoxybenzaldehyde, and 1,2-benzenedicarboxylic acid bis (2-methylpropyl) ester, respectively.

From the value of *q* and the initial permethrin concentration, the kinetic parameters including *K*_i_, *K*_s_, and *q*_max_ for substrate inhibition model were established to be 4.7912 mg·L^−1^, 367.2165 mg·L^−1^ and 0.0454 h^−1^, respectively using nonlinear regression analysis by Matrix Laboratory (MATLAB) software (Version 7.8). As shown in Figure 5A, the greater permethrin removal efficiency was achieved at the lower initial concentrations of permethrin (<100 mg·L^−1^), indicating that microbial activity could be inhibited by higher concentrations of permethrin. Similar results were observed in other pyrethroid-degrading bacteria (Chen et al., 2013, 2015; Xiao et al., 2015). The critical inhibitor concentration (*S*_m_) was determined to be 41.9453 mg·L^−1^ by calculating the square root of *K*_i_^*^ *K*_s_. The determination coefficient *R*^2^ was 0.9913, indicating that the experimental data were fitted well with the Luong model.

3-Phenoxybenzenemethanol is a major metabolite from the degradation of SPs (Maloney et al., 1988). In this study, strain ZH-14 tolerated and degraded 3-phenoxybenzenemethanol up to a concentration of 500 mg·L^−1^. Degradation of this compound by the same strain that degraded SPs was of great importance because 3-phenoxybenzenemethanol is not only refractory to biodegradation but also limits the accelerated degradation of the SPs due to its antimicrobial activities (Laffin et al., 2010). Previous report that transformation of 3-phenoxybenzenemethanol (20 mg·L^−1^) by *Pseudomonas fluorescens* SM-1 was achieved in the presence of Tween 80 (0.05% [vol/vol]), but this strain was unable to grow or transform 3-phenoxybenzenemethanol in the absence of the primary carbon source (Maloney et al., 1988). Specially, strain ZH-14 was capable of utilizing 3-phenoxybenzenemethanol as the sole carbon source.

The decrease in the specific degradation rate following an increase in the initial 3-phenoxybenzenemethanol concentration suggests that this chemical may act as a partial inhibitor to strain ZH-14. The kinetic curve of 3-phenoxybenzenemethanol degradation at different initial concentrations is shown in Figure 4B. *K*_i_, *K*_s_, *S*_m_, and *q*_max_ for 3-phenoxybenzenemethanol inhibition model were determined to be 6.3064 mg·L^−1^, 176.9207 mg·L^−1^, 33.4026 mg·L^−1^, and 0.0366 h^−1^, respectively. As shown in Figure 5B, when the initial concentration of 3-phenoxybenzenemethanol was lower than 33.4026 mg·L^−1^, *q* value was gradually increased. At higher concentrations, inhibition by 3-phenoxybenzenemethanol became substantial and *q* value was proportionally decreased in a dosage dependent manner. It reveals that the chemical degradation activity of strain ZH-14 may be partially inhibited at a high concentration of 3-phenoxybenzenemethanol but may not lead to a complete repression.

### Metabolites identification

To explore the mechanism of permethrin degradation by *A. baumannii* ZH-14, the metabolites from permethrin degradation were extracted and confirmed by HPLC and GC-MS. In this study, the metabolites identified by mass spectrometry analysis were matched with authentic standard compounds from the National Institute of Standards and Technology (NIST, USA) library database. Four main degradation products were identified along with the permethrin degradation, on the basis of similarity of their fragment retention times (RT) and molecular ions to those of corresponding authentic compounds in the NIST library database (Figure S3). The chemical structures, RT and characteristic ions of the mass spectra (m/z) are summarized in Table 3. In the sample of 12 h, a significant peak was detected with RT of 29.070 min, showing a characteristic mass fragment [M^+^] at m/z 391 with a major fragment ion at m/z 183, which was characterized as permethrin and named compound A. Along with the disappearance of compound A, three new compounds B, C, and D were detected, respectively. Compound B, with RT of 18.979 min showed a prominent protonated molecular ion at m/z 200, was confirmed as 3-phenoxybenzenemethanol. Compounds C and D, with RT of 16.959 and 20.627 min presented molecular ions at m/z 198 and 149, were identified as 3-phenoxybenzaldhyde and 1,2-benzenedicarboxylic acid bis (2-methylpropyl) ester, respectively (Figure 5). Among these identified metabolites, compounds C and D were detected for the first time in the biodegradation pathway of permethrin. It is noteworthy that all these intermediate products were transient and they disappeared finally. No persistent accumulative metabolite was observed after 72 h.

**Table 3:** Chromatographic properties of metabolites of permethrin during degradation by strain ZH-14.

**Compound**	**RT (min)**	**m/z**	**Chemical structural formula in NIST library**	**Name**
A	29.070	391	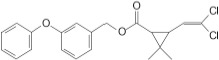	Permethrin
B	18.979	200	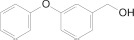	3-Phenoxybenzenemethanol
C	16.959	198	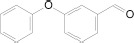	3-Phenoxybenzaldehyde
D	20.627	279	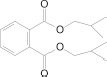	1,2-benzenedicarboxylic acid bis (2-methylpropyl) ester

**Figure 6:**
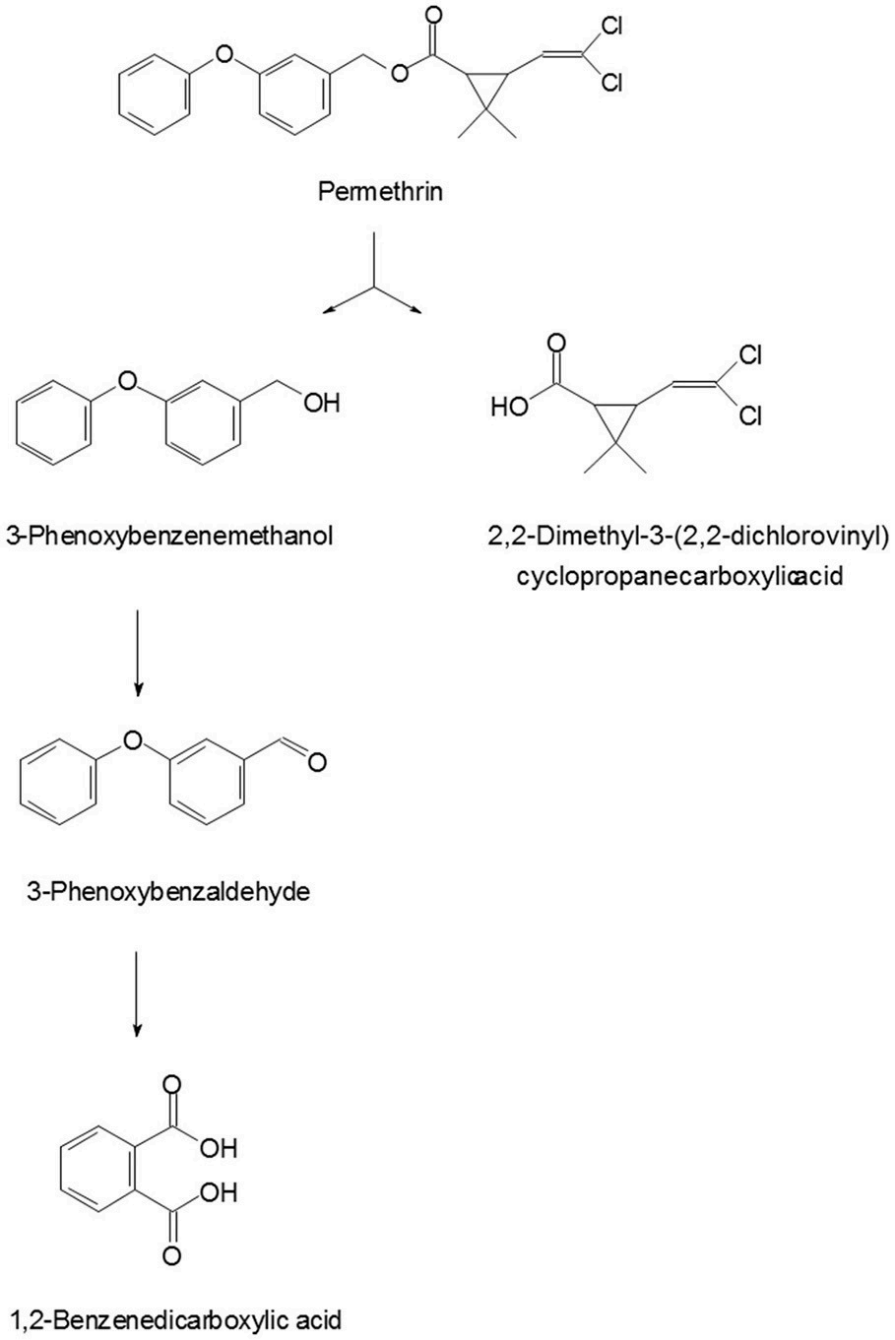
Proposed pathway for degradation of permethrin in strain ZH-14.

**Figure 7:**
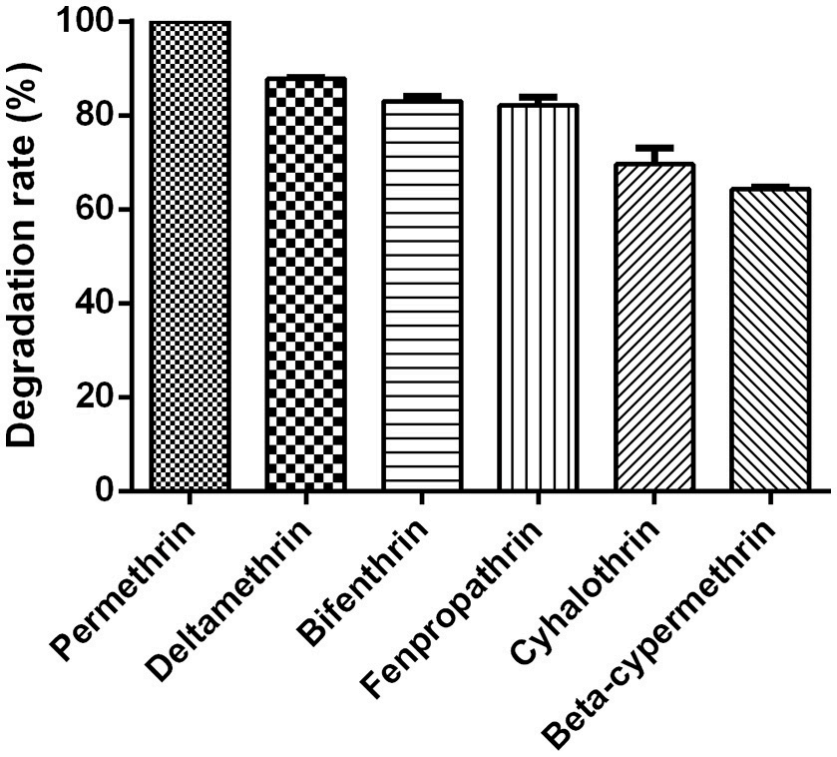
Degradation rates of various SPs by strain ZH-14 within 72 h. Data represent mean values of three replicates with standard deviation.

A new metabolic pathway of permethrin in strain ZH-14 was proposed based on analysis of the chemical structures of permethrin and the metabolites (Figure 6). As shown in Figure 7, permethrin was initially degraded by hydrolysis of its ester linkage to yield 2,2-dimethyl-3-(2,2-dichlorovinyl) cyclopropanecarboxylic acid and 3-phenoxybenzenemethanol which was further transformed to 3-phenoxybenzaldhyde via redox reaction. 3-Phenoxybenzaldhyde was then subject to diaryl cleavage to form 1,2-benzenedicarboxylic acid which may undergo subsequent metabolism by cleavage of aromatic ring. Permethrin was detoxicated by strain ZH-14 without any persistent accumulative product, thus, we deduce the bacterial strain may harbor a complete metabolic pathway for degradation and metabolism of permethrin.

Hydrolysis is the initial step in the degradation pathway of permethrin by strain ZH-14, consistent with previous findings of Maloney et al. (1988). This chemical contains an ester bond structure in molecular (Figure 1), which is susceptible to attack via hydrolysis. Similar results have been observed in other microorganisms involved in SPs degradation (Tallur et al., 2008; Zhang et al., 2011; Chen et al., 2013; Xiao et al., 2015). Significantly, in addition to hydrolysis of ester bond, strain ZH-14 further transformed permethrin by cleavage of the diaryl bond, resulting in complete detoxification of this pesticide.

### Degradation kinetics of various SPs

The degradation capacity for different SPs by strain ZH-14 is presented in Figure 7. Strain ZH-14 utilized permethrin, deltamethrin, bifenthrin, fenpropathrin, cyhalothrin, and beta-cypermethrin as the growth substrates with the degradation rates of 100, 87.9, 83.0, 82.2, 69.6, and 63.8% within 72 h, respectively. This particular strain is capable of degrading a broad spectrum of SPs with high degradation efficiency, suggesting strain ZH-14 may be an ideal microorganism for bioremediation of the pyrethroid-contaminated environments. SPs contain cyclopropane carboxylic acid moieties linked to aromatic alcohols through a central ester bond (Chen et al., 2014). In our study, permethrin was preferentially transformed by strain ZH-14, which could be attributed to the reason that the cyano group is more toxic and persistent to microbial degradation (Cycoń and Piotrowska-Seget, 2016). Moreover, strain ZH-14 transformed not only permethrin but also other SPs, which is rarely seen in other degraders (Maloney et al., 1988; Tallur et al., 2008; Zhang et al., 2011; Zhai et al., 2012).

Kinetic data indicated that the degradation process followed the first-order kinetic model [Equation (3)], so this model was used to investigate the biodegradation kinetics of various SPs. Furthermore, the theoretical half-life (*t*_1/2_) values of various SPs were calculated by Equation (4). The degradation kinetic parameters of degradation of various SPs by strain ZH-14 are tabulated in Table 4. The degradation coefficients *R*^2^ of permethrin, deltamethrin, bifenthrin, fenpropathrin, cyhalothrin, and beta-cypermethrin were 0.9311, 0.9434, 0.9695, 0.9562, 0.9602, and 0.9494, respectively, indicating that the degradation data are all fitted well with the first-order kinetic model. The degradation process was characterized by a *k* ranging from 0.0160 to 0.0038 h^−1^. The high specific degradation rate further demonstrated strain ZH-14 is highly effective in degrading SPs. With strain ZH-14, the *t*_1/2_ values were determined to be 18.2–43.3 h, which are remarkably shortened as compared to these SPs in the environment with the reported *t*_1/2_ values varying from 17 to 600 days (Laskowski, 2002).

**Table 4:** Kinetic parameters of degradation of SPs by strain ZH-14.

**SPs**	**Regression eq**	***k* (h^−1^)**	***t*_1/2_ (h)**	***R*^2^**
Permethrin	*C_*t*_* = 54.4942e^−0.0381*t*^	0.0381	18.2	0.9311
Deltamethrin	*C_*t*_* = 54.3266e^−0.0260*t*^	0.0260	26.7	0.9434
Bifenthrin	*C_*t*_* = 53.3340e^−0.0252*t*^	0.0252	27.5	0.9695
Fenpropathrin	*C_*t*_* = 53.3487e^−0.0267*t*^	0.0267	26.0	0.9562
Cyhalothrin	*C_*t*_* = 52.0893e^−0.0194*t*^	0.0194	35.7	0.9602
Beta-cypermethrin	*C_*t*_* = 51.1242e^−0.0160*t*^	0.0160	43.3	0.9494

### Biodegradation of permethrin in soils

To explore the ability of *A. baumannii* ZH-14 in bioremediation of permethrin-contaminated soils, bioaugmentation with strain ZH-14 was carried out in sterile and nonsterile soils, respectively. The degradation process was investigated using the first-order kinetic model adapted from Cycoń et al. (2014). The kinetic parameters are presented in Table 5. After bioaugmentation with strain ZH-14, ~86.4% of initial added permethrin was eliminated in nonsterilized soils within 9 days, and the degradation process was characterized by a rate constant (*k*) of 0.1640 day^−1^ with a *t*_1/2_ of 4.2 days (Table 5). Moreover, no obvious lag phase was observed during the 9-day experiment. Whereas, in controls with autochthonous microflora, this activity decreased only by 31.0% giving its *t*_1/2_ of 16.3 days. Previous reports on other SPs have demonstrated that pure isolates which can degrade them in culture conditions also perform their degradation in soils (Chen et al., 2011b, 2012, 2014; Cycoń et al., 2014).

**Table 5:** Kinetic parameters of degradation of permethrin in sterile and nonsterile soils inoculated with strain ZH-14.

**Treatment**	**Regression eq**	***k* (day^−1^)**	***t*_1/2_ (days)**	***R*^2^**
Sterile soils + permethrin	*C_*t*_* = 49.9139e^−0.0246*t*^	0.0246	28.2	0.9827
Nonsterile soils + permethrin	*C_*t*_* = 50.4569e^−0.0425*t*^	0.0425	16.3	0.9949
Sterile soils + permethrin + ZH-14	*C_*t*_* = 52.2506e^−0.1412*t*^	0.1412	4.9	0.9253
Nonsterile soils + permethrin + ZH-14	*C_*t*_* = 52.3811e^−0.1640*t*^	0.1640	4.2	0.9606

In case of sterilized soils with strain ZH-14 about 85.1% of the initial dose of permethrin was removed after 9 days of experimentation and this disappearance process was characterized a *k* of 0.1412 day^−1^ with a *t*_1/2_ of 4.9 days. In comparison with the control treatment without strain ZH-14, the abiotic degradation rate with naturally occurring attenuation only reached 21.4% in 9 days giving its *t*_1/2_ of 28.2 days. These results confirmed the observations made by Maloney et al. (1988) and Wang et al. (2009) showing that microbial degradation is the main mechanism of permethrin dissipation in the environment whereas its abiotic breakdown is less important. Very interesting, our results indicated the degradation rate of permethrin by strain ZH-14 in nonsterilized soils was higher than that observed in sterilized soils, which contrasted with previous findings of Cycoń et al. (2014) who reported *S. marcescens* strains DeI-1 or DeI-2 introduced into sterile soils showed a higher degradation potential for deltamethrin removal (16–26 or 5–17%) than those observed in nonsterile soils. Our results revealed that naturally occurring microorganisms strongly enhanced the strain ZH-14 to degrade permethrin, which might be due to the reason that the introduced strain ZH-14 and indigenous microflora may have a synergistic effect on metabolization of this xenobiotic compound, as described previously by Chen et al. (2011b). However, the degrading microorganisms isolated from environment usually fail to degrade pollutants in soils due to the low activity of these isolates caused by abiotic and biotic stresses, thus additional treatments are needed to enhance biodegradation (Zhang et al., 2012; Liu et al., 2015). In our study, strain ZH-14 efficiently degraded permethrin in soils without any other further treatment, suggesting that this strain has promising potentials and advantages as a bioremediation organism in removing permethrin residues from various environments.

## Conclusions

A newly isolated strain *A. baumannii* ZH-14 was capable of utilizing a wide range of SPs as food sources, thus giving it an exceptional ability to colonize ecological niches where nutrients are limited. The optimum conditions for enhanced biodegradation were determined to be temperature 30°C, pH 7.0 and an inocula size of 0.2 g·L^−1^, resulting in 100% degradation of permethrin (50 mg·L^−1^) within 72 h. This is the first report of complete degradation of permethrin by a pure isolate. This particular strain degraded permethrin up to a concentration of 800 mg·L^−1^ with a *q*_*max*_, *K*_*s*_, *K*_*i*_, and *S*_m_ of 1.14 d^−1^ of 0.0454 h^−1^, 4.7912 mg·L^−1^, 367.2165 mg·L^−1^, and 41.9453 mg·L^−1^, respectively. Strain ZH-14 degraded permethrin via a novel metabolic pathway with the formation of 3-phenoxybenzenemethanol, 3-phenoxybenzaldhyde, and 1,2-benzenedicarboxylic acid bis (2-methylpropyl) ester as the main intermediate metabolites, which were further transformed without any persistent accumulative product, indicating that the bacterial strain may harbor a complete metabolic pathway for degradation and metabolism of permethrin. Bioaugmentation of permethrin-contaminated soils with strain ZH-14 significantly enhanced the removal rate of permethrin, and its half-life was remarkably reduced from 16.3–28.2 days to 4.2–4.9 days in different soils, revealing that this strain is a potential and efficient candidate for bioremediation of pyrethroid-contaminated terrestrial and aquatic environments.

## Author contributions

SC designed the experiments; HZ, HW, YF, XF, and SC performed the experiments; HZ, LL, XF, and SC performed data analysis; LZ provided scientific expertise; HZ, HW, LL, YF, XF, and SC wrote the manuscript.

### Conflict of interest statement

The authors declare that the research was conducted in the absence of any commercial or financial relationships that could be construed as a potential conflict of interest.
