# Supplementary material for: Kinetics and Novel Degradation Pathway of Permethrin in Acinetobacter baumannii ZH-14
Source: Front Microbiol. 2018 Feb 2;9:98. doi: 10.3389/fmicb.2018.00098 (PMC5801723; doi:10.3389/fmicb.2018.00098)
Supplement: Supplementary file 1 [file DataSheet1.DOC]

**Supplemental files**

**Kinetics and novel degradation pathway of permethrin in *Acinetobacter baumannii* ZH-14**

Hui Zhan, Huishan Wang, Lisheng Liao, Yanmei Feng, Xinghui Fan, Lianhui Zhang, Shaohua Chen^*^

State Key Laboratory for Conservation and Utilization of Subtropical Agro-bioresources, Guangdong Province Key Laboratory of Microbial Signals and Disease Control, Integrative Microbiology Research Centre, South China Agricultural University, Guangzhou 510642, China

**Keywords:** Permethrin; Degradation pathway; Kinetics; *Acinetobacter baumannii*; Bioremediation

**Running title:** Catabolism of permethrin

^*^**Corresponding author:** Shaohua Chen, South China Agricultural University, No 483 Wushan Road, Tianhe District, Guangzhou 510642, China; Fax: +86-20-85280292; Tel: +86-20-85288229; E-mail: [shchen@scau.edu.cn](mailto:shchen@scau.edu.cn)

**Table S1** Box-Behnken experimental design matrix and the response of dependent variable for permethrin degradation by strain ZH-14

|  |  |  |  | Response (*Y*_1_) |
| --- | --- | --- | --- | --- |
| Run | *X*_1_ | *X*_2_ | *X*_3_ | Permethrin residues (%) |
| 1 | -1 | -1 | 0 | 12.1 |
| 2 | -1 | 1 | 0 | 16.4 |
| 3 | 1 | -1 | 0 | 8.7 |
| 4 | 1 | 1 | 0 | 10.5 |
| 5 | 0 | -1 | -1 | 11.6 |
| 6 | 0 | -1 | 1 | 5.3 |
| 7 | 0 | 1 | -1 | 13.8 |
| 8 | 0 | 1 | 1 | 10.9 |
| 9 | -1 | 0 | -1 | 10.4 |
| 10 | 1 | 0 | -1 | 7.6 |
| 11 | -1 | 0 | 1 | 5.2 |
| 12 | 1 | 0 | 1 | 3.6 |
| 13 | 0 | 0 | 0 | 0 |
| 14 | 0 | 0 | 0 | 0 |
| 15 | 0 | 0 | 0 | 0 |

*X*_1_ refers to pH: -1 (6.0), 0 (7.0), +1 (8.0); *X*_2_ refers to temperature: -1 (25 °C), 0 (30 °C), +1 (35 °C); *X*_3_ refers to inocula: -1 (0.05 g·L^-1^), 0 (0.15 g·L^-1^), +1 (0.25 g·L^-1^).

**Table S2** The biochemical tests by Biolog Microbial Identification System

| Characteristics | Results (24h) | Characteristics | Results (24h) |
| --- | --- | --- | --- |
| D-Maltose | ＋ | Dextrin | – |
| Stachyose | – | D-Trehalose | ＋ |
| Gentiobiose | ＋ | Sucrose | ＋ |
| D-Cellobiose | ＋ | D-Turanose | ＋ |
| D-Raffinose | – | α-D-Lactose | – |
| β-Methyl-D-  Glucoside | ＋ | D-Melibiose | – |
| D-Salicin | none | N-Acetyl-D-  Glucosamine | ＋ |
| N-Acetyl-β-D-  Mannosamine | ＋ | N-Acetyl-D-  Galactosamine | none |
| N-Acetyl  Neuraminic Acid | – | 1% NaCl | ＋ |
| 4% NaCl | ＋ | 8% NaCl | – |
| α-D-Glucose | ＋ | D-Mannose | ＋ |
| D-Fructose | ＋ | D-Galactose | ＋ |
| 3-Methyl Glucose | – | D-Fucose | ＋ |
| L-Fucose | ＋ | L-Rhamnose | ＋ |
| Inosine | – | 1% Sodium | ＋ |
| Fusidic Acid | – | D-Serine | － |
| D-Sorbitol | ＋ | D-Mannitol | ＋ |
| pH 6 | ＋ | pH 5 | － |
| D-Arabitol | ＋ | myo-Inositol | ＋ |
| Glycerol | – | D-Glucose-6-PO4 | – |
| D-Fructose-6-PO4 | – | D-Aspartic Acid | none |
| D-Serine | ＋ | Troleandomycin | ＋ |
| Rifamycin SV | ＋ | Minocycline | ＋ |
| Gelatin | － | Glycyl-L-Proline | ＋ |
| L-Alanine | ＋ | L-Arginine | ＋ |
| L-Aspartic Acid | ＋ | L-Glutamic Acid | ＋ |
| L-Histidine | ＋ | L-Pyroglutamic Acid | － |
| L-Serine | ＋ | Lincomycin | ＋ |
| Guanidine HCl | ＋ | Niaproof 4 | ＋ |
| Pectin | ＋ | D-Galacturonic Acid | ＋ |
| Mucic Acid | ＋ | Quinic Acid | ＋ |
| D-Saccharic Acid | － | Vancomycin | ＋ |
| Tetrazolium Violet | ＋ | Tetrazolium Blue | ＋ |
| p-Hydroxy-  Phenylacetic Acid | － | Methyl Pyruvate | ＋ |
| D-Lactic Acid  Methyl Ester | ＋ | L-Lactic Acid | ＋ |
| Citric Acid | ＋ | α-Keto-Glutaric Acid | ＋ |
| D-Malic Acid | ＋ | L-Malic Acid | ＋ |
| Bromo-Succinic  Acid | ＋ | Nalidixic Acid | － |
| Lithium Chloride | ＋ | Potassium Tellurite | ＋ |
| Tween 40 | ＋ | γ-Amino-Butryric Acid | ＋ |
| α-Hydroxy-Butyric Acid | ＋ | β-Hydroxy-D,L-  Butyric Acid | ＋ |
| α-Keto-Butyric Acid | ＋ | Acetoacetic Acid | ＋ |
| Propionic Acid | ＋ | Acetic Acid | ＋ |
| Formic Acid | － | Aztreonam | ＋ |
| Sodium Butyrate | ＋ | Sodium Bromate | － |
| L-Galactonic  Acid Lacone | ＋ | D-Gluconic Acid | ＋ |
| D-Glucuronic Acid | ＋ | Glucuronamide | ＋ |

＋, tested positive; －, tested negative


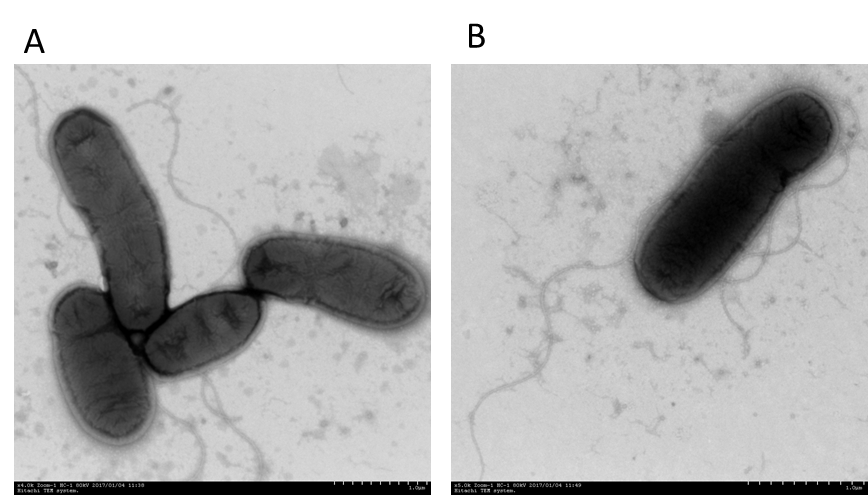


**Figure S1** Morphological characteristics of strain ZH-14 under Hitachi TEM System. A: 4000×; B: 5000×


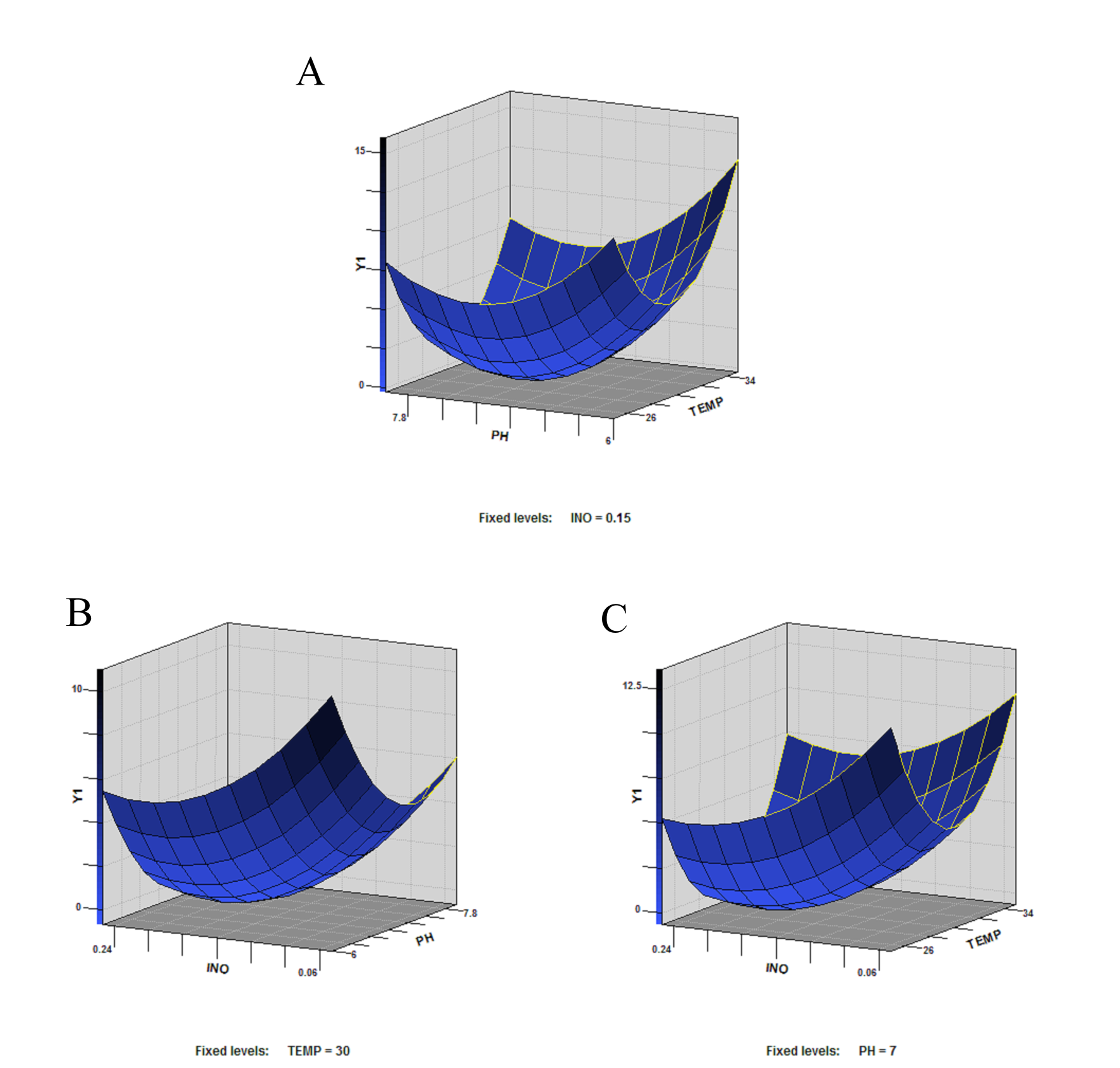


**Figure S2** Response surface plots showing the interactive effects on permethrin degradation by strain ZH-14. (A) the effect of pH and temperature on permethrin biodegradation while fixing the value of inocula size at a zero level (0.15 g·L^-1^); (B) the effect of inocula size and pH on permethrin biodegradation while fixing the value of temperature at a zero level (30 °C); (C) the effect of inocula size and temperature on permethrin biodegradation while fixing the value of pH at a zero level (7.0).

A

B

C

D

**Figure S3** The mass spectra of permethrin biodegradation products reported in the National Institute of Standards and Technology (NIST, USA) library database. (A) permethrin; (B) 3-phenoxybenzenemethanol; (C) 3-phenoxybenzaldehyde; (D) 1,2-benzenedicarboxylic acid bis (2-methylpropyl) ester.
